# Assessment of methods used to determine the safety of the topical insect repellent *N,N*-diethyl-*m*-toluamide (DEET)

**DOI:** 10.1186/1756-3305-7-173

**Published:** 2014-06-03

**Authors:** Vanessa Chen-Hussey, Ron Behrens, James G Logan

**Affiliations:** 1 Department of Disease Control, Faculty of Infectious and Tropical Diseases, London School of Hygiene & Tropical Medicine, Keppel Street, London WC1E 7HT, UK; 2 Department of Clinical Research, Faculty of Infectious and Tropical Diseases, London School of Hygiene & Tropical Medicine, Keppel Street, London WC1E 7HT, UK

**Keywords:** *N,N*-diethyl-*m*-toluamide, Insect repellents, Safety, Toxicity

## Abstract

*N,N*-diethyl-*m*-toluamide (DEET) has been registered for commercial use as an insect repellent for over five decades, and is used widely across the world. Concerns over the safety of DEET first emerged during the 1980s after reports of encephalopathy following DEET exposure, particularly in children. However, the role of DEET in either the illness or deaths was and remains purely speculative. In response to these cases a number of reviews and investigations of DEET safety were carried out. Here we examine the methods used and information available to determine the safety of DEET in humans. Animal testing, observational studies and intervention trials have found no evidence of severe adverse events associated with recommended DEET use. Minor adverse effects noted in animal trials were associated with very large doses and were not replicated between different test species. The safety surveillance from extensive humans use reveals no association with severe adverse events. This review compares the toxicity assessment using three different models to define the risk assessment and safety threshold for DEET use in humans and discusses the clinical consequences of the thresholds derived from the models.

The theoretical risks associated with wearing an insect repellent should be weighed against the reduction or prevention of the risk of fatal or debilitating diseases including malaria, dengue, yellow fever and filariasis. With over 48 million European residents travelling to regions where vector borne diseases are a threat in 2009, restricting the concentration of DEET containing repellents to 15% or less, as modelled in the 2010 EU directive, is likely to result in extensive sub-therapeutic activity where repellents are infrequently applied. Future European travellers, as a consequence of inadequate personal protection, could potentially be at increased risk of vector borne diseases. Risk assessments of repellents should take these factors into account when setting safe limits.

## Introduction

*N,N*-diethyl-*m*-toluamide (DEET) is amongst the most effective and widely used of insect repellents [1]. DEET was one of the first synthetic repellents, initially developed by the United States Department of Agriculture and then used by the military. It was registered for use by the general public in 1957, and remains the most widely used repellent with an estimated 30% of the US population using insect repellent in 2000 [2]. In 2011, a sample survey for the EPA, showed that US consumers bought insect repellent on average 2.87 times per year, and 3.49 times if there were children in the family [3]. Given the greater awareness of vector borne diseases such as West Nile virus, tick-borne encephalitis and Lyme disease, insect repellent use is likely to increase, and sales reports show that sales of insect repellents increased 28% between 2001 and 2003 [4]. There are now a range of synthetic and naturally derived repellent compounds, and of these, Icaridin and PMD have been shown to provide effective protection, however, due to its long history DEET still has the strongest body of evidence for efficacy [5]. Another synthetic repellent, IR3535 has shown reduced efficacy against *Anopheles* mosquitoes and as such is not recommended for travellers to endemic areas [5]. In testing of new actives ingredients, 20% DEET is the gold standard for comparison [6] and shows efficacy against a wide variety of blood-feeding organisms: from mosquitoes and black flies to ticks, mites and even land leeches [7].

Concerns over the safety of DEET first emerged in the 1980s after reports of encephalopathy following DEET exposure, particularly in children [8,9]. Although the role of DEET in these cases was purely speculative, they prompted a number of investigations and a number of reviews of the safety of DEET.

**Figure 1:**
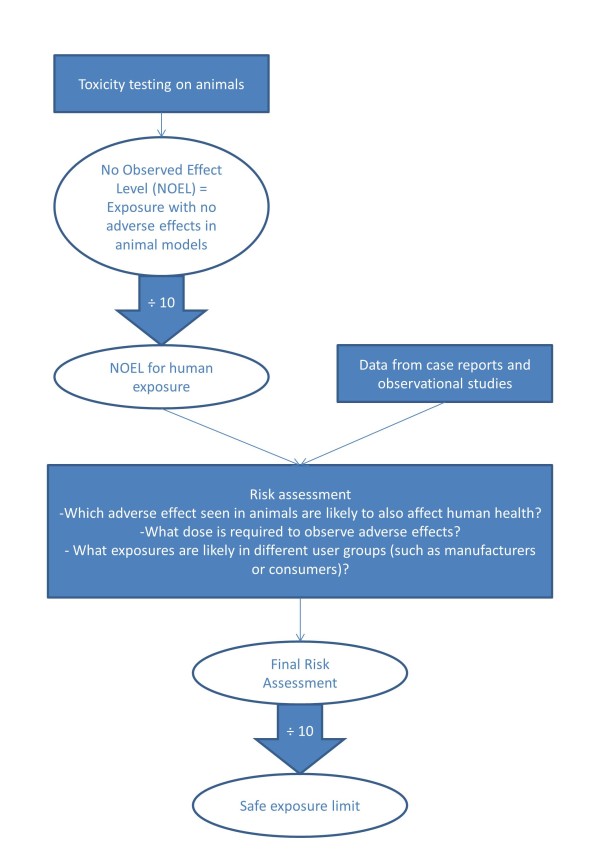
The processes involved in creating a safe exposure assessment of any chemical.

The process of undertaking a comprehensive safety risk assessment of a chemical requires a four stage review [10]. The first stage is hazard identification, which identifies the capacity of the chemical to generate adverse effects in humans. The second is a dose–response test to establish a relationship between dose and the incidence of adverse events. The third includes toxicity assessment data from animal or human exposure to define a final safe exposure limit in humans (Figure 1). Much of the data used to assess the safety of DEET relies on animal studies, with only a single observational trial of DEET use in pregnancy so far carried out on humans. The final stage is a risk assessment that combines the safe exposure limits with likely levels and routes of exposure.

### Toxicity studies of DEET in animals

Animal toxicity testing provides basic data where human exposure information is inadequate or unavailable. Most tests only collect information on obvious toxicity effects such as changes in body weight, without defining the mechanisms of toxicity. The United States Environmental Protection Agency (USEPA) carried out a series of experiments in 1998 as part of a DEET re-registration process [11]. The majority of studies revealed minor effects of DEET and different NOEL, depending on the exposure route (Table 1). The experiments of most interest involve dermal exposures to DEET, including a 90-day dermal toxicity study in rats using exposures up to 1000 mg/kg body weight/day. At the greatest exposure male rats were observed to develop renal lesions and a decreased body weight, and both sexes showed increased liver weights. As renal lesions were observed in all males at every exposure level, the study was unable to establish a no observed effect level (NOEL). Detailed investigations revealed that the renal lesions were unique to male rats and not relevant to human health [11]. A similar study using micropigs, found no changes in mortality, body weight, blood biochemistry, gross pathology or organ weights due to DEET exposure up to 1000 mg per kg body weight per day [11].

**Table 1:** **Results of toxicity testing of DEET on animals reported to the USEPA**[11]

**Test type**	**Animal**	**Outcome**
Acute oral toxicity	Rat	Oral LD_50_ = 2170–3664 mg/kg
		Toxicity category III: Slightly toxic and slightly irritating
Acute dermal toxicity	Rabbit	Dermal LD_50_ = 4280 mg/kg
		Toxicity category III: Slightly toxic and slightly irritating
Acute inhalation toxicity	Rat	Inhalation LD50 = 5.95 mg/kg
		Toxicity category IV: Practically non-toxic and not an irritant
Subchronic (90 day) oral toxicity	Rat	Renal effects found in exposed male rats of two of three strains tested so no NOEL established.
Subchronic (90 day) oral toxicity	Hamster	NOEL: 61 mg/kg/day
		LEL: 305 mg/kg/day
Subchronic (90 day) dermal toxicity	Rat	Renal lesions in all male rats treated so no NOEL established
Subchronic (90 day) dermal toxicity	Micropigs	NOEL: 1000 mg/kg/day
Chronic (2 year) toxicity	Rats and dogs	NOEL: 100 mg/kg/day
		LEL: 400 mg/kg/day
		No carcinogenic effects
Chronic (78 weeks) toxicity	Mice	NOEL: 500 mg/kg/day
		LEL: 1000 mg/kg/day
		No carcinogenic effects
Reproductive toxicity	Rats	NOEL: 250 mg/kg/day (highest dose tested)
Developmental and maternal toxicity	Rats	NOEL: 250 mg/kg/day
		LEL: 750 mg/kg/day
Developmental toxicity	Rabbits	NOEL: 325 mg/kg/day (Highest dose tested)

### Studies and case reports of DEET safety in humans

Studies in humans have clear advantages as they avoid the errors when extrapolating results from animal studies. In humans, secondary risk factors such as smoking and diet can also be considered. Testing involving animals will largely be drawn from homogenous populations with similar susceptibility to the chemical being tested, whereas human populations will mostly contain a wide range of individuals with varying susceptibility. Observational studies and case reports form the basis of most human toxicological studies. Case reports, although highly subjective and sometimes anecdotal, can be useful in identifying rare associated effects such as unique reactions or association with rare diseases. Observational studies, not easily done, do not allow for any control of assignment of study subjects to either group or the levels of exposure and controlling for confounding factors. Randomised trials using human subjects provide the most rigorous data on safety, but for ethical and safety reasons these trials are rare in toxicological investigations.

Although no human studies have been carried out specifically to assess the safety of DEET, a randomised trial of DEET used to prevent malaria transmission amongst pregnant women in Thailand did assess health outcomes in participants [12]. The women were all past their first trimester and when recruited to the trial were monitored for the duration of the pregnancy at weekly ante-natal visits. Their babies were then monitored monthly until six months old and then every three months until one year. No significant differences were found between the DEET treatment group and control group in weight, height, head and arm circumferences, and neurologic performance in newborns. Similarly, infants followed to one year showed no significant difference in developmental delay, death, weight, height, head circumference and arm circumference between those whose mothers had used DEET and non-users. Neither were there any differences in the occurrences of neurological or gastrointestinal effects in the women. Skin warming, a heating sensation on the skin, was significantly more common in the DEET group. DEET was not detected in urine, however, it was detected in the cord blood from four (8%) of the women indicating that DEET is able to cross the placenta. The ability of DEET to cross the placenta is confirmed in a survey of pesticides in cord blood amongst mothers in New Jersey [13]. DEET was detectable in the blood and cord blood of all women analysed, although no significant effects on birth weight, head circumference or birth length were found, but a borderline association between higher DEET levels in the cord blood and higher abdominal circumference was noted. At present such trials are rare and limited, so there is no data for the safety of DEET in the first trimester, on the effects of ante-natal DEET exposure beyond one year follow up.

During the mid-1980s there were six reported cases of encephalopathy following exposure to DEET, all in girls aged 1–8 years which resulted in three deaths [8]. However, it is unclear whether exposure to DEET was the real cause of the seizures or deaths. The first case involved a 3 and a half year old girl who developed seizures, and it was reported that for the two weeks prior to the illness DEET had been applied to her clothes, bedding and skin resulting in an estimated exposure of 0.14 ml per kg body weight per day [14]. One of the fatal cases involved a six year old girl [15] who had applied DEET ‘extensively’. However, in this case a history of similar episodes without DEET exposure led to the hypothesis that the girl may have had undiagnosed ornithine carbamyl transferase deficiency. In this case medical opinion was that, although DEET may have precipitated the episode, the cause of death may have been one of the medicines administered during her stay in hospital. A further case was of an 8 year old girl who developed a rash on areas where DEET had been ‘copiously’ applied in the previous 4 days [16]. She then suffered seizures before making full recovery. The authors reporting the case believed that, in contrast to previous reports, this case was a true hypersensitive reaction to DEET.

Ten years later there had been a further eight cases of nervous system toxicity following DEET exposure. No gender bias was found across all fourteen cases, but all but one involved children under 8 years [17]. The prevalence of encephalopathy during this age range is higher as is the use of DEET use and encephalopathy, therefore it is not surprising to find an apparent association in some cases [18]. Despite the term ‘DEET-induced encephalopathy’ in some case reports [19], no link to dose or mechanistic pathway has been demonstrated between the use of DEET and the occurrence of encephalopathy. Although DEET might be causally associated in these cases, often there is a lack of data on the actual concentrations and exposures involved. A full analysis of these cases is not intended in this review, as the subject has been ably covered by other authors [9,17]. An analysis was made of over 9,000 calls relating to DEET exposure that were placed to American Poison Control Centres from 1985–9 [20]. Almost 90% were treated solely at home and 80% of those referred to a health centre were discharged after initial examination suggesting mild or short-lived symptoms. The severity of symptoms was found to be more closely related to the type of exposure with inhalation or contact with eyes causing greater symptoms, than the concentration of DEET or the age or gender of the patient. A second review of over 20,000 calls between 1993–7 found consistent results [21].

A DEET Registry was created to collate information on severe adverse neurological or systemic events associated with DEET use in a standardised way in order to evaluate any potential causal relationship [9]. Two hundred and forty-two cases that had been referred to the DEET Registry between 1995 and 2001 were assessed by qualified nurses and followed up after one year. It was found that children were not disproportionately represented amongst cases. Children made up 41% of DEET cases in this analysis compared to 65% of any calls to Poisons Centres between 1983 and 2009 [22]. However, compared to adults in the analysis, children were more likely to have experienced seizures and 71% of seizure patients were children. This is not a remarkable finding, however, as seizures are more common in children than adults [23]. No relationship was evident between DEET concentration and severity of outcome, but as with the case reports of seizures described above, estimating actual DEET exposure is difficult given the variation in self-administered doses.

Even when allowing for a large factor of underreporting, the incidence of 14 reported cases of DEET-associated encephalopathy since 1957 is small when considered against the context of an estimated 200 million applications of DEET worldwide each year [2]. Some individuals can develop allergic responses to DEET, which result in serious reactions through even small exposures [24]. However, for adult consumers, and based on these observational studies, there is no basis in the observational data for concerns over the safety of DEET.

### Risk assessment of DEET

Results from animals need to be extrapolated to be relevant to human health. It is standard practice to take 10% of NOEL from animal data to account for differences between animal and human metabolism and then a further adjustment to 10% of that dose (100^th^ the original dose), to create a safe maximum exposure limit [10]. However, as this approach can lead to a large correction factor, it is acknowledged that risk assessments resulting in unacceptable exposures should be further examined to ensure the findings are sensible and that a more refined assessment is not required [25].

Antwi *et al.* carried out a risk assessment of DEET [26] using both the results of USEPA toxicity testing and those of Schoenig *et al.* in which DEET was only administered orally [11,27]. To estimate risk to human health NOELs were set at 200 mg/kg body weight/day for acute exposures, 300 mg/kg body weight/day for subchronic exposures and 100 mg/kg body weight/day for chronic exposures. A survey of repellent use by men, women and children found that the dose applied did not vary with age, gender or the actual DEET concentration [28]. The average dose applied in a single application was 3.7 g. Antwi *et al.* then used this to model the level of DEET exposure associated with the use of 5%, 25% and 40% DEET repellent products. None of the estimated exposures were equal or greater than the NOELs. However, a single 3.7 g application of a 40% DEET product is above 1% of the NOEL for all age groups and a single application of 25% is above 1% of the NOEL for children under 17 years old. The 1% of the NOEL, as established from animal testing, is recognised as the safe exposure limit. This therefore means a 40% DEET product is qualified as unsafe for human use but is based on rat experimental data, where animals at higher dose exposures developed non-specific weight loss and male renal abnormalities.

The USEPA committee, however, considered the effects observed in rats at 1000 mg per kg body weight per day as so modest and the dosage so high, that it did not form sufficient grounds for conducting a quantitative risk assessment. They did not ,therefore, define a minimum dose of DEET for daily use.

The 2010 EU directive, using the same data source as the USEPA recommends an acceptable exposure level (AEL) for repeated use of 8.2 mg DEET per kg body weight per day [29]. This dose is equivalent to a single application of 3.3 g of a 15% DEET formulation to an adult weighing 60 kg. The directive has used the standard 1% correction shown in Figure 1 as well as an 82% correction for different absorption rates between animals and humans to achieve this final safety margin. This assessment did not reference the different outcomes and findings of the USEPA study.

All three studies have used the same toxicity data to determine safe exposure levels, but only the USEPA considered a full risk assessment was not necessary. Based on the modest effects of DEET shown in the human toxicity data, it is questionable as to whether a risk assessment should be done. The decision about whether a chemical should be given a full risk assessment is an important step as often chemicals with low toxicity do not require this assessment. The different conclusions, based on the same data, demonstrate the difficulties and uncertainties involved in modelling toxicity.

### Synergistic effects of DEET with insecticides

Illnesses reported by service personnel returning from the Gulf War have been linked to synergistic effects of DEET used alongside permethrin (an insecticide impregnated into clothing) and pyridostigmine bromide (a prophylactic agent against the effects of nerve gas). In toxicity studies using animals, rats exposed to combinations of DEET and pyridostigmine bromide had greater than expected mortality, but lower than expected mortality was observed in rats exposed to combinations of DEET and permethrin [11]. Pyridostigmine bromide alone has been shown to impair rat behavioural performance, but combined with DEET leads to further loss in performance [30]. As an anti-nerve gas agent, few people would be expected to be exposed to pyridostigmine bromide. Therefore, it is possible to control combined exposure in these select group without prohibiting the use of DEET. The combination of DEET and permethrin is a more likely combination in use in the general population with products such as insecticide treated bed nets and clothing. In vitro and animal experiments show that DEET is a cholinesterase inhibitor [31], and in combination with permethrin has measurable effects indicating DNA damage and oxidative stress in rats, and also increases the release of rat brain mitochondrial cytochrome C and disrupts the blood–brain barrier (BBB) in rats [32]. Further work is required to determine the mechanism that could lead to adverse effects for human health using doses and applications that are relevant to DEET use by travellers.

### Implications for disease transmission

The use of repellents as personal bite protection is recommended to travellers for protection against arthropod borne diseases in tropical countries. However, mostly due to concerns over cost rather than long term exposure, it is not recommended to people living in vector-borne disease endemic countries. Although there have been relatively few studies that have measured disease outcomes associated with the use of repellents alone, and those that have been published have resulted in variable results in protection, there is evidence that topical repellents can reduce vector borne disease transmission including malaria [33,34] as well as pediculosis, sandfly fever and scrub typhus [35]. The efficacy of repellents, in part, relies on the efficacy of the active ingredient, the longevity (how long it lasts) and user compliance. Undoubtedly, DEET is the most effective and long-lasting providing the concentration is high enough and it is well-formulated. In laboratory testing different concentrations have been shown to provide different protection times. For example, in one study, formulations of DEET below 20% provided complete protection for only 20 minutes, whereas in comparable tests, those between 20-50% provided complete protection for 3 hours [36]. Bearing in mind the likely effect of sweating and rubbing off on clothes in hot climates, the level of DEET on the skin is likely to reduce rapidly in this environment. In another study, people who exercise after applying a repellent based soap have been found to have 10-30% less protection from bites compared to those who had not exercised [37]. Therefore, to achieve high levels of protection and maintain this over a practical time period before having to reapply is essential for any repellents to protect against disease transmission in travellers. The suggested application rate of 8.2 mg DEET per kg body weight per day, equates to a single application of 2.46 g of a 20% DEET product for an adult. The application volume or weight of a DEET product, based on the standard application used in repellency testing of 1.67 ml/mg/cm^2^ required to cover 2 arms would be 5.7 ml/g product (18% surface area). A 15% concentration product would contain 855 mg DEET and a 50% product would contain 2850 mg of DEET. These DEET levels are above the Directives maximum 492 mg per day (for a person weighing 60 kg) and would not allow repeated application or application to other parts of the body sufficient to provide long lasting protection against most vectors of disease. In field testing of 20-35% DEET formulations, investigators have used up to 6 ml to protect exposed limbs, usually forearms, lower legs and feet [38], but sometimes only the lower legs [39,40].

## Conclusions

Risk assessments of chemical exposures rely on exposure and toxicity assessments which both involve a margin of error. Risk assessments use a conservative estimate of exposure to describe the lowest safe estimates of toxicity. To ensure low-risk chemicals are not subjected to lengthy assessments a tiered approach is normally used [25]. With DEET assessments, this is the point at which there is a difference in approach between researchers. The standard risk assessment takes the toxicity profile in rats, applies a conversion factor to apply the data to humans and another to establish a safe exposure level. This blind approach finds normal exposure DEET products above 15% leads to excessive exposure. In contrast, other researchers considered the observed adverse effects in rats (reduced food consumption and decreased weight gain) has no relevance to human risk and therefore do not complete any further risk assessment.

Biting arthropods transmit an array of vector-borne pathogens that cause diseases including malaria, dengue, Lyme disease, filariasis, onchocerciasis and West Nile virus. These diseases have a significant human health impact and many have no specific prophylaxis, treatment or vaccines. Therefore, insect repellents are important in reducing or preventing these insect borne infections. DEET is the most effective and widely used insect repellent with an estimated 200 million annual applications worldwide. With over 48 million European residents travelling annually to regions where vector borne diseases are a threat [41], restricting the concentration of DEET containing repellents to 15% or less, as modelled in the 2010 EU directive based on toxicity estimates from NOELS in animals, is likely to result in extensive sub-therapeutic activity where repellents are infrequently applied [5,42]. Comparisons between marketed repellents show that formulation as well as DEET concentration will greatly effect longevity of protection [43], therefore, although not yet available, low DEET repellents could eventually provide a long-lasting protection. Future European travellers, as a consequence of inadequate personal protection, are likely to be at increased risk of vector borne diseases. It is therefore essential that any risk assessment of the toxicity of DEET needs to be balanced by its benefit in avoiding disease and extensive global data on the widespread use of DEET and reported human toxicity.

Policy recommendation on DEET use needs to balance the available toxicity data, and its historical safety record, against the risks of vector borne diseases. Conventional risk assessment methodology leads to a recommendation to reduce the use of higher concentrations of DEET, but fails to recognise the disease avoiding benefits of DEET. A pragmatic approach along with future research to describe toxicity thresholds in humans are the way forward.

## Abbreviations

DEET: *N,N*-diethyl-*meta*-toluamide; EU: European Union; NOEL: No observed effect Level; USEPA: United States Environmental Protection Agency.

## Competing interests

JL is the Director and VCH is a Research Scientist at *arctec* the Arthropod Control Product Test Centre at the London School of Hygiene & Tropical Medicine. *arctec* provides impartial commercial testing services on insect repellent efficacy.

## Authors’ contributions

VCH carried out the literature review and wrote the initial draft. RB reviewed the manuscript and provided additional sections on traveller health. JL reviewed the manuscript and gave final approval. All authors read and approved the final version of the manuscript.
